# Supplementary material for: Molecular simulations of enzymatic phosphorylation of disordered proteins and their condensates
Source: Nat Commun. 2025 May 19;16:4649. doi: 10.1038/s41467-025-59676-4 (PMC12089381; doi:10.1038/s41467-025-59676-4)
Supplement: Supplementary file 1 — Supplementary Information [file 41467_2025_59676_MOESM1_ESM.pdf]

Supplementary Information

Emanuele Zippo[1,2], Dorothee Dormann[2,3], Thomas Speck[4], Lukas S. Stelzl[2,3,5,*]

1 Institute of Physics, Johannes Gutenberg University Mainz, Mainz, Germany
2 Institute of Molecular Physiology, Johannes Gutenberg University Mainz, Mainz, Germany
3 Institute of Molecular Biology (IMB), Mainz, Germany
4 Institute for Theoretical Physics IV, University of Stuttgart, Stuttgart, Germany
5 KOMET1, Institute of Physics, Johannes Gutenberg University Mainz, Mainz, Germany
***** email: `lstelzl@uni-mainz.de`

Supplementary Figures

Markov-state models of simulations of chemically-driven dynamics

Dependence of rates from MC move attempt rate

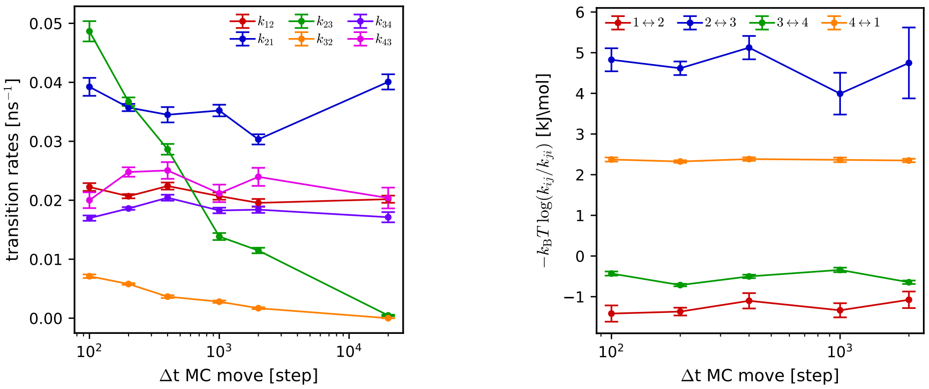

Supplementary Figure 1: Transition probabilities T_{ij} at lag time 10 MSM steps (10^5 MD steps) (left) and logarithm of the rate ratios (right) for each transition for different choice of MC move attempt rate for the case of reactive Ser 403 and $\Delta\mu_\mathrm{P} = -5$ kJ mol^{-1}. Data represent mean values +/- SEM estimated by bootstrapping 100 samples from the 15 μs long discretized trajectories.

Dependence of rate ratios from $\Delta\mu_\mathrm{P}$

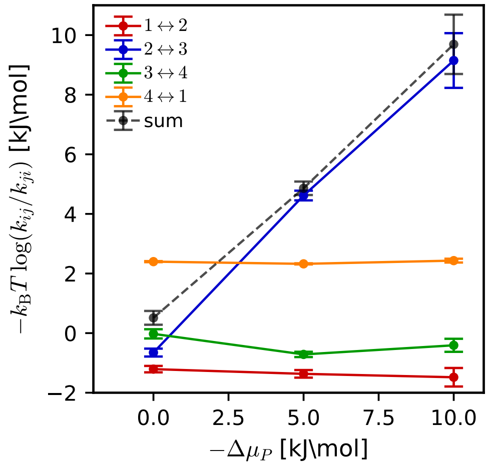

Supplementary Figure 2: Logarithm of the rate ratios for each transition at different $\Delta\mu_\mathrm{P}$. Data represent mean values +/- SEM estimated by bootstrapping 100 samples from the 15 μs long discretized trajectories.

CK tests and implied timescales

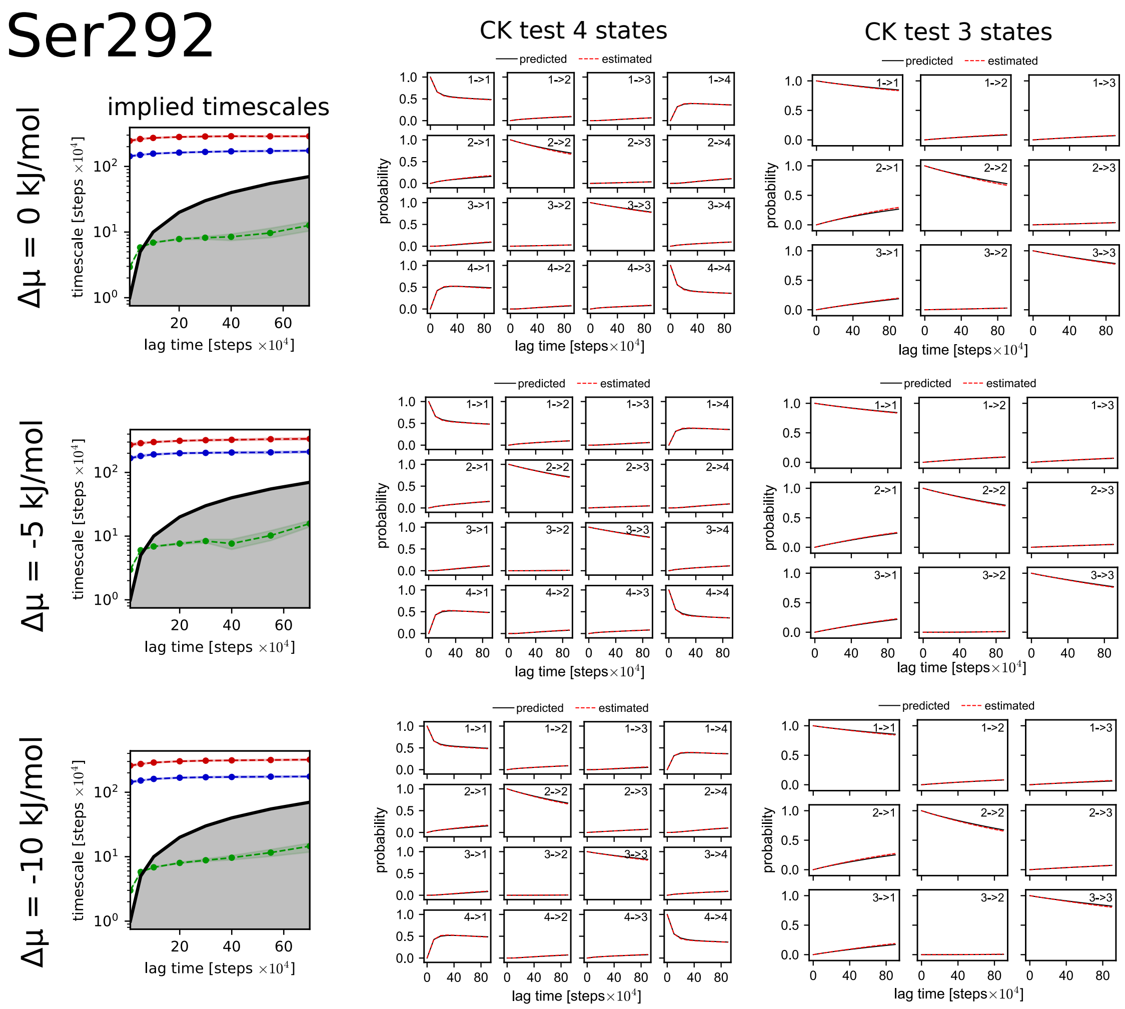

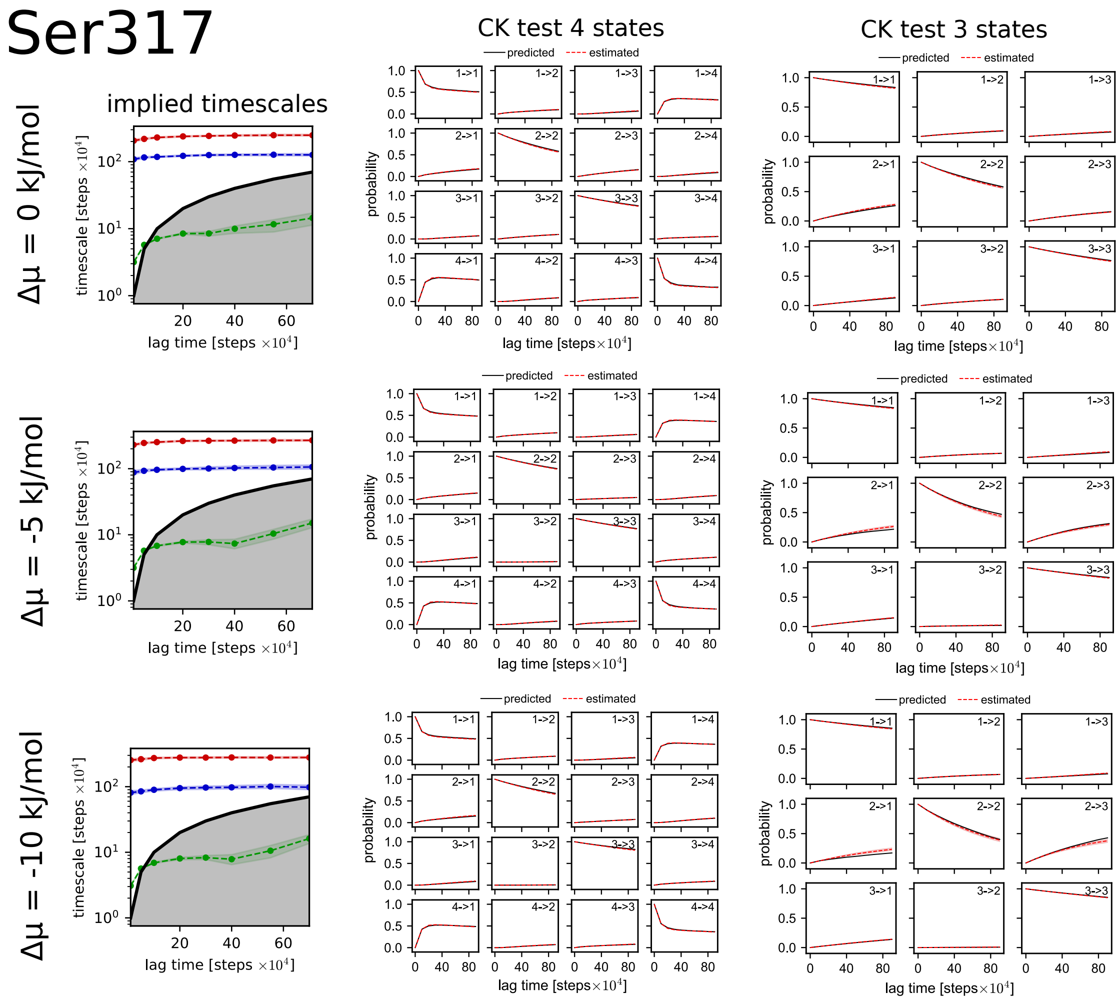
Ser317
implied timescales
CK test 4 states
CK test 3 states
Δμ = 0 kJ/mol
Δμ = -5 kJ/mol
Δμ = -10 kJ/mol
timescale [steps ×10⁴]
lag time [steps ×10⁴]
probability
predicted
estimated

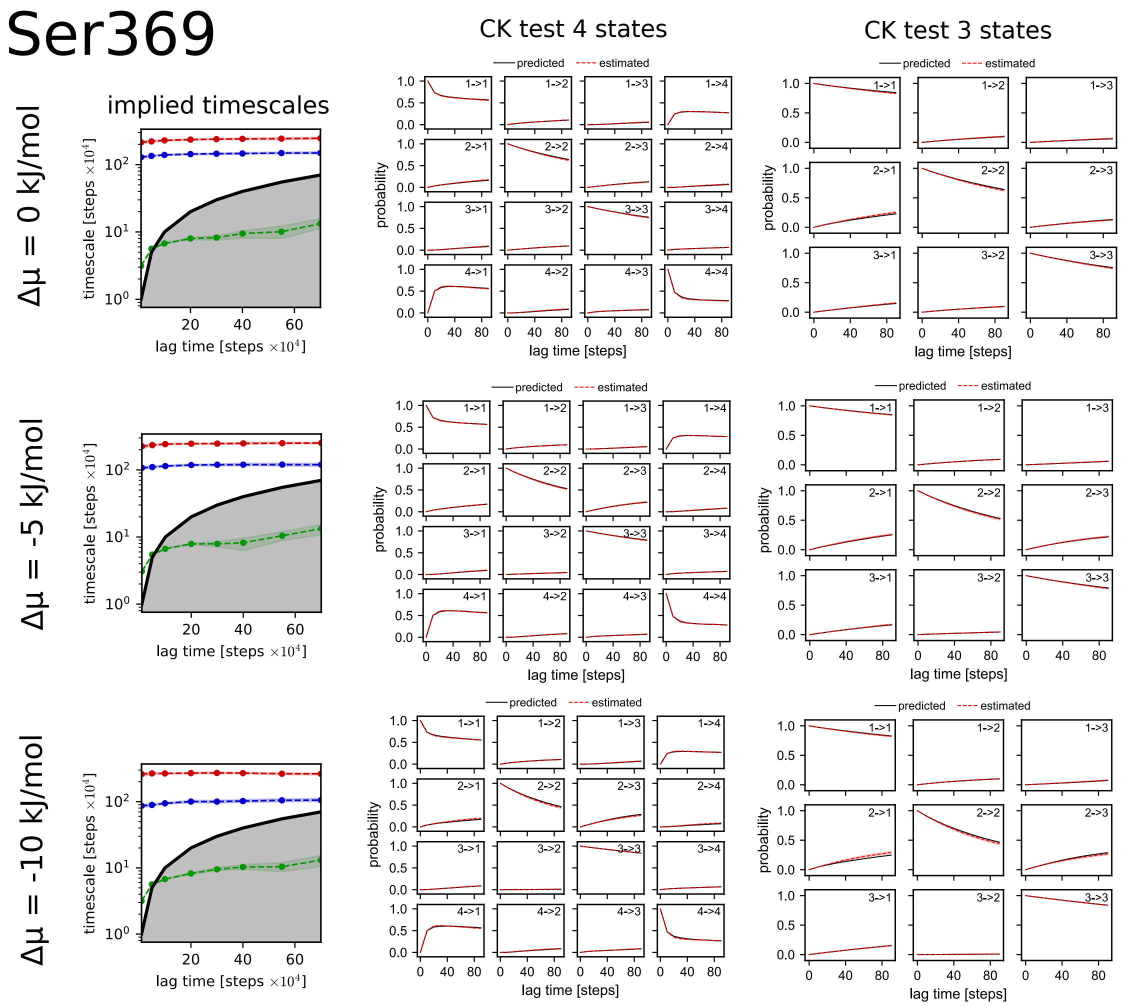
Ser369
implied timescales
CK test 4 states
CK test 3 states
Δμ = 0 kJ/mol
Δμ = -5 kJ/mol
Δμ = -10 kJ/mol
timescale [steps ×10⁴]
lag time [steps ×10⁴]
predicted
estimated
probability
lag time [steps]

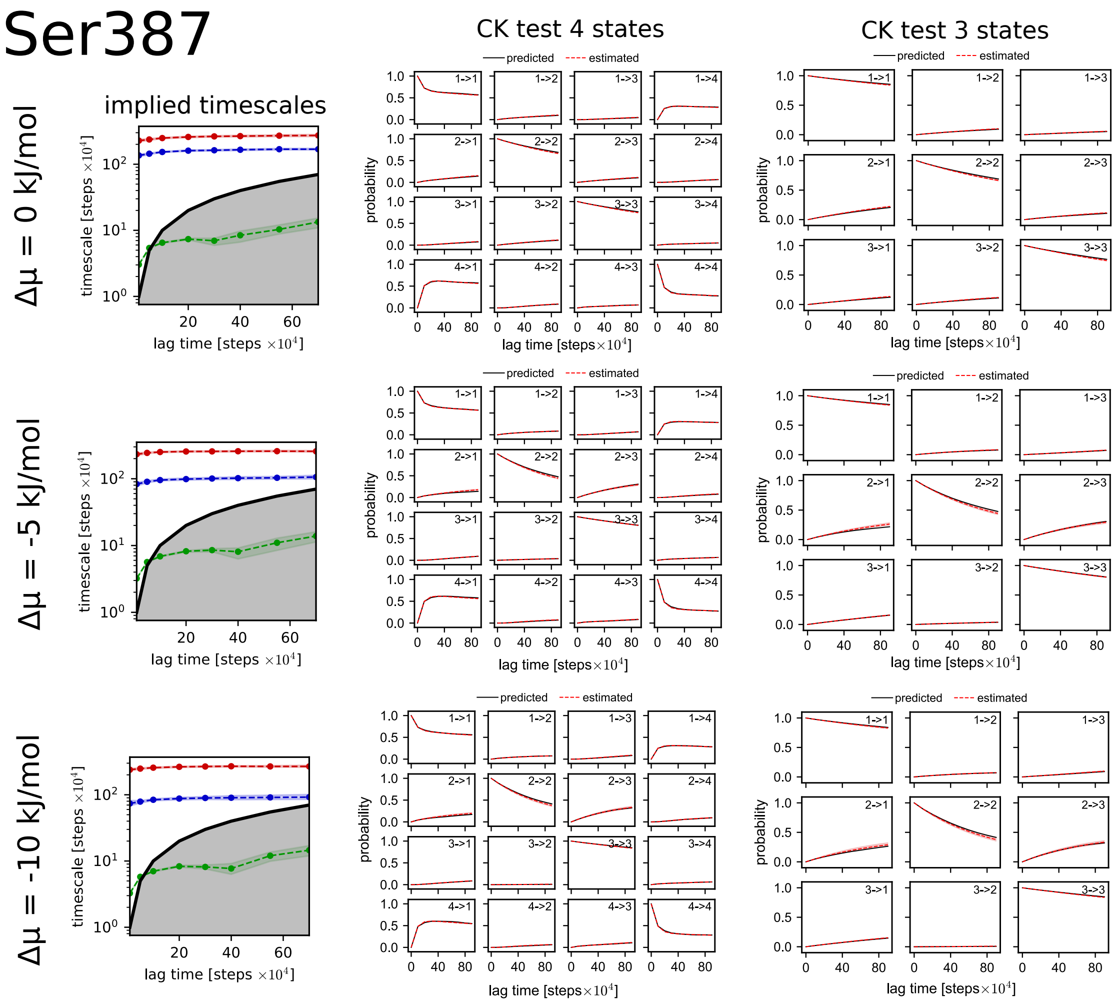

Ser387
implied timescales
CK test 4 states
CK test 3 states
predicted
estimated
Δμ = 0 kJ/mol
Δμ = -5 kJ/mol
Δμ = -10 kJ/mol
timescale [steps ×10⁴]
lag time [steps ×10⁴]
probability

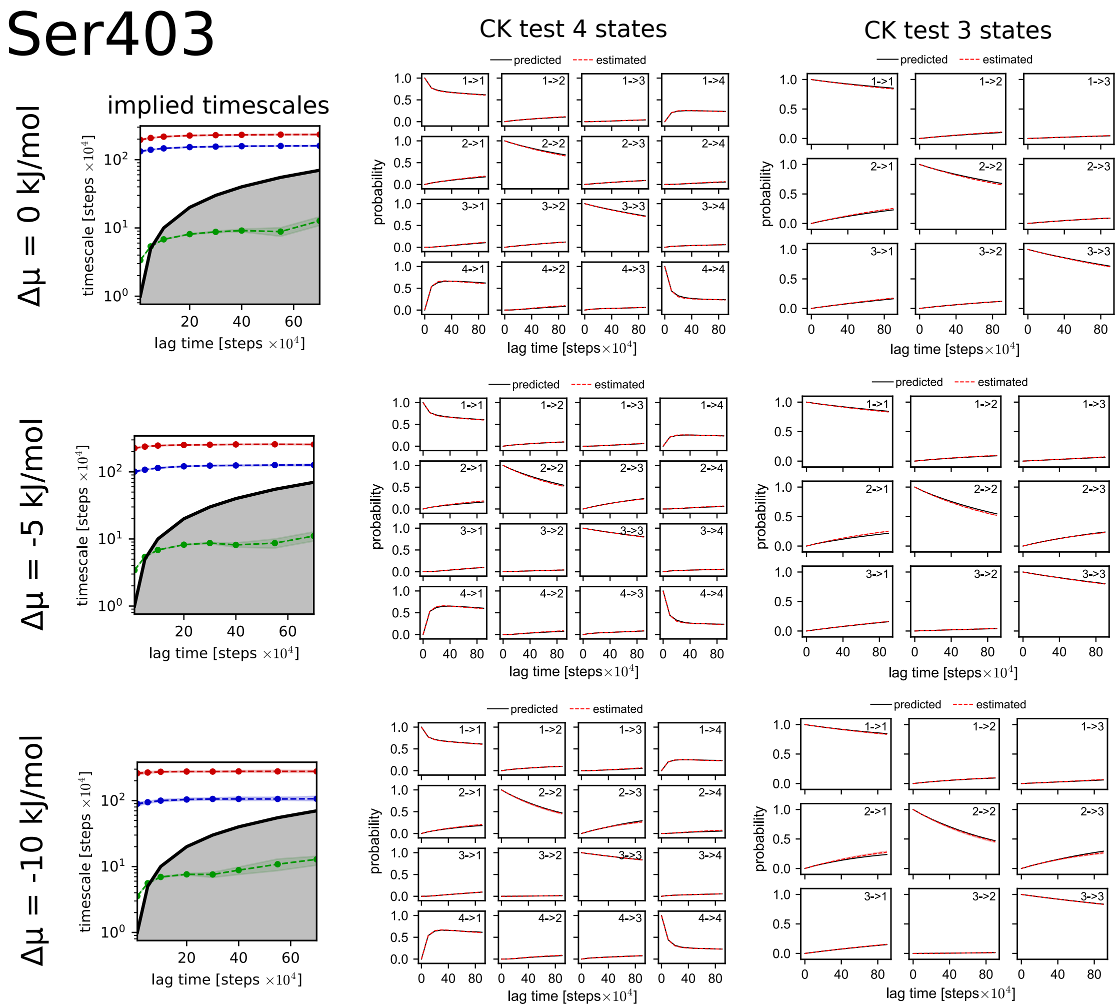
Ser403
implied timescales
CK test 4 states
CK test 3 states
Δμ = 0 kJ/mol
Δμ = -5 kJ/mol
Δμ = -10 kJ/mol
predicted
estimated
timescale [steps ×10⁴]
lag time [steps ×10⁴]
probability

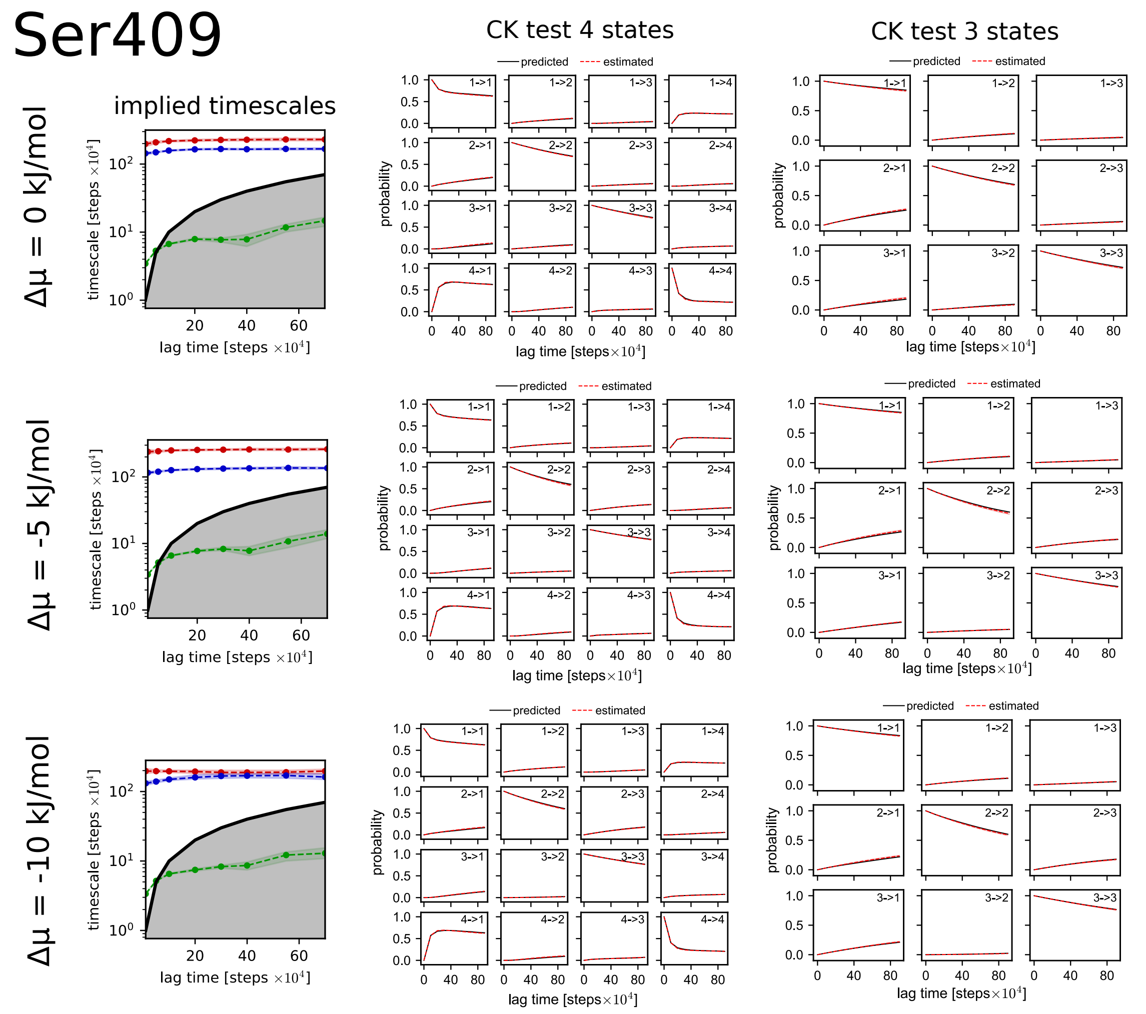

Supplementary Figure 3: Implied timescales (shaded areas are SEM from 100 bootstrapped samples) and CK test (shaded areas are 95% confidence intervals from 100 bootstrapped samples) for every validation simulation. We estimated $\Delta\mu_{\mathrm{cycle}}$ also with a 3-state MSM, merging together state 1 and 4 into the new state 1. We report the CK test also for the 3-state MSM case.

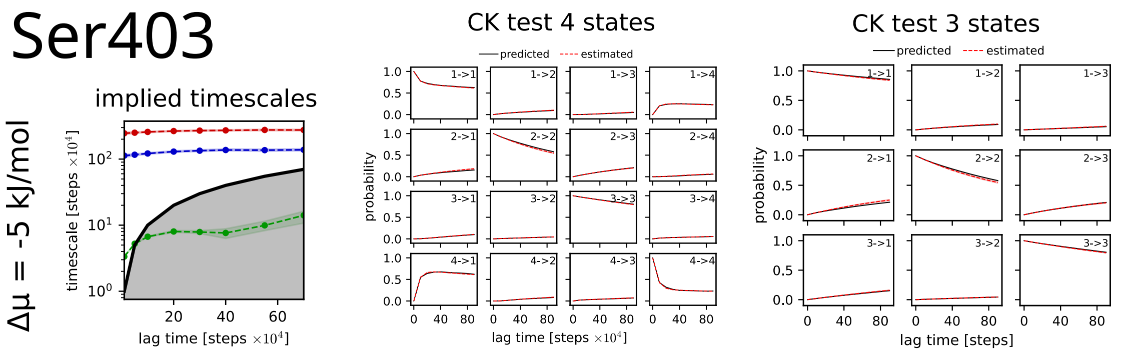

Supplementary Figure 4: Implied timescales (shaded areas are SEM from 100 bootstrapped samples) and CK test (shaded areas are 95% confidence intervals from 100 bootstrapped samples) for Ser403 and $\Delta\mu_{\mathrm{P}} = -5\ \mathrm{kJ\ mol}^{-1}$ using a version of VAMPnet with 4260 input distances. We estimated $\Delta\mu_{\mathrm{cycle}}$ also with a 3-state MSM, merging together state 1 and 4 into the new state 1. We report the CK test also for the 3-state MSM case.

C-terminus of TDP-43 is preferentially phosphorylated

CK1δ sequence and charges

a)

b)

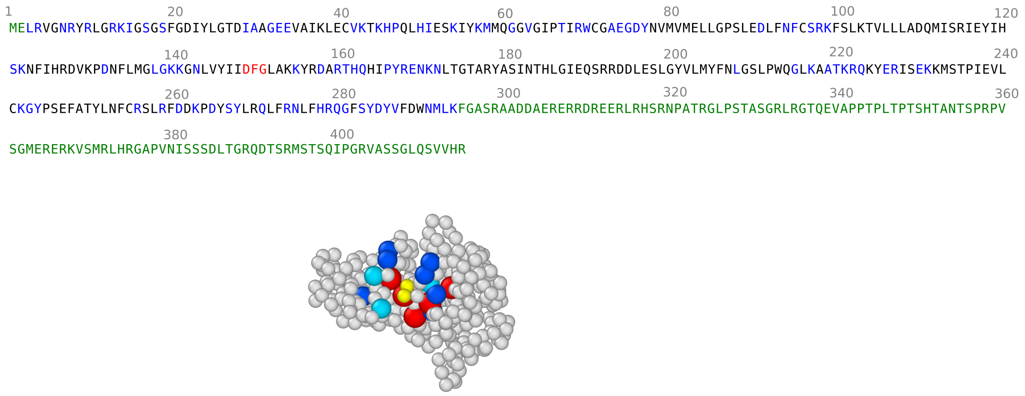

Supplementary Figure 5: **(a)** Full-length CK1δ sequence. Surface residues in blue, IDR residues in green, active site residues in red. **(b)** CK1δ with colored charged residue close to the active site (blue +e, light blue His, red -e, yellow active site).

TDP-43 LCD contacts per residue

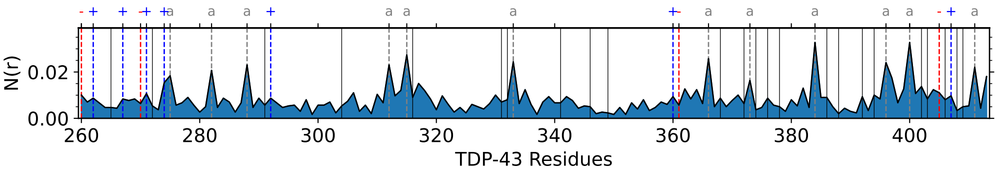

Supplementary Figure 6: Contact frequency for each residue of WT TDP-43 LCD with folded CK1δ from single-chain simualtion at equilibrium. The ticks on top show the position of the charged (blue '+' positive, red '-' negative), aromatic (grey 'a') and methionine (green 'M') residues.

Phosphorylation MC step acceptance probability

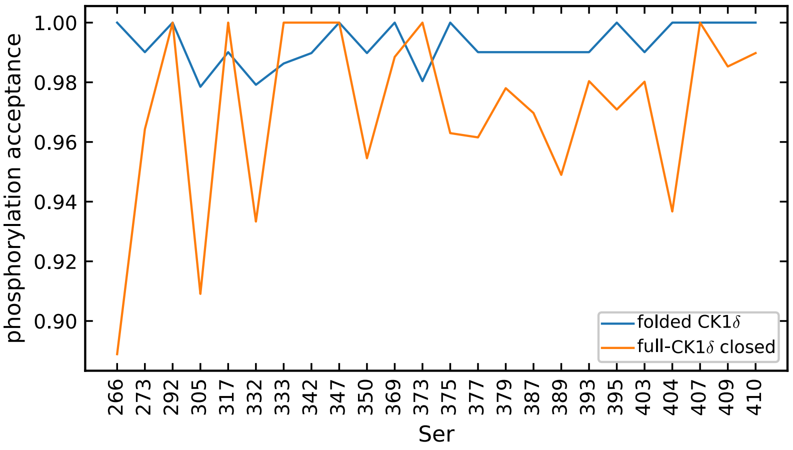

Supplementary Figure 7: Acceptance ratio for phosphorylation step for simulations with CK1δ folded-domain (blue) and full-length CK1δ closed (orange).

Comparison with CALVADOS3 force field

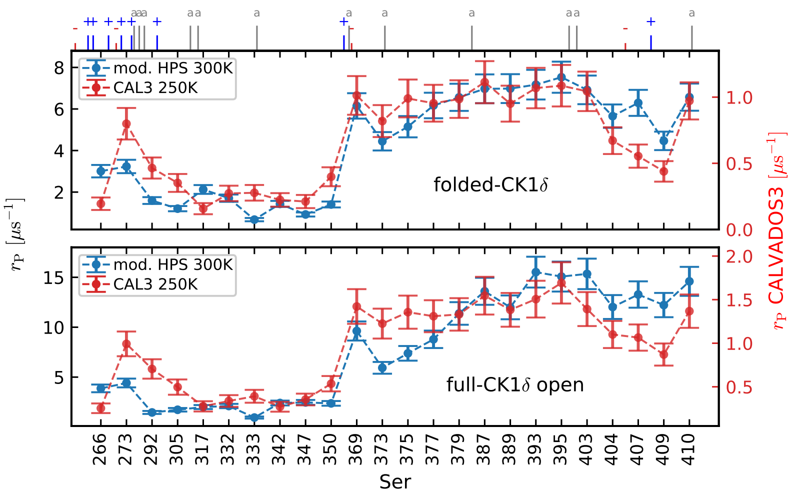

Supplementary Figure 8: Mean values +/- SEM of r_P from 100 replicas with modified HPS force field at 300K (blue) and from 50 replicas with CALVADOS3 force field at 250K (red, right y-axis). The ticks on top show the position of the charged (blue '+' positive, red '-' negative) and aromatic (grey 'a') residues.

Effect of TDP-43 helical region in single chain simulations

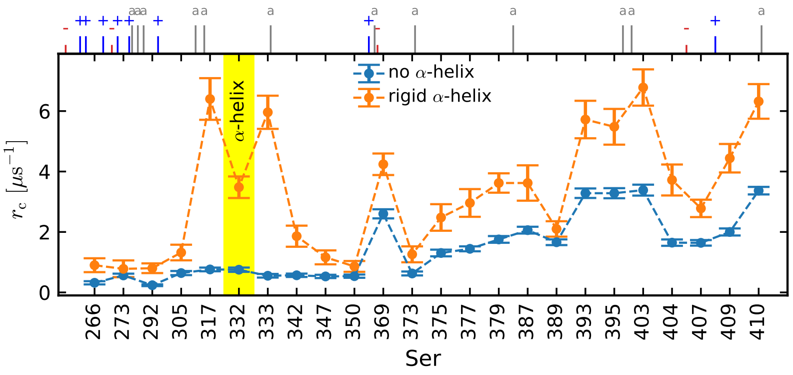

Supplementary Figure 9: Mean values +/- SEM of r_c from 10 replicas with TDP-43 with helical region fixed as rigid body (orange) and from 30 replicas without preserved helix (blue). The yellow area highlights the position of the helical region. The ticks on top show the position of the charged (blue '+' positive, red '-' negative) and aromatic (grey 'a') residues.

Prior phosphorylation alters TDP-43 phosphorylation dynamics

Phosphorylation process fit

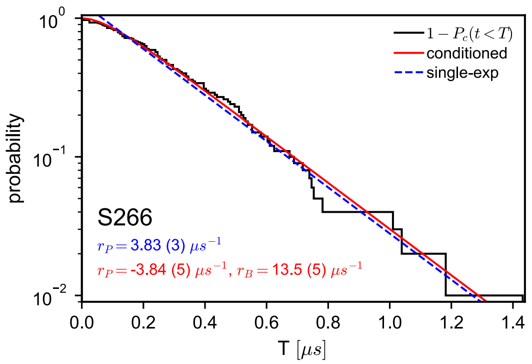

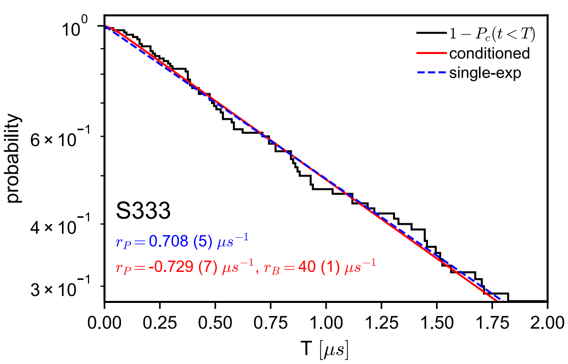

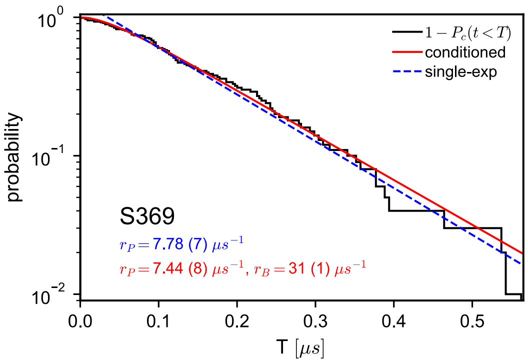

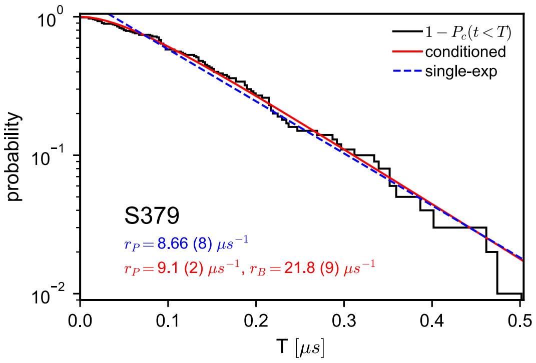

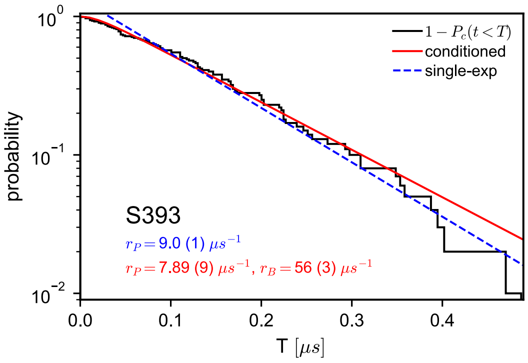

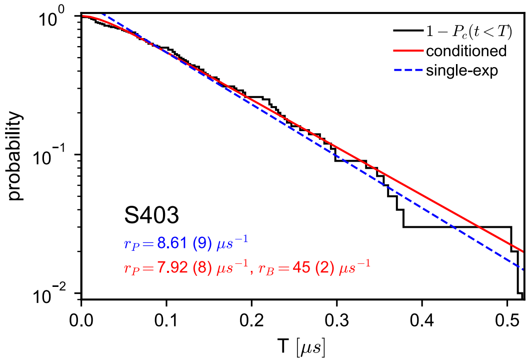

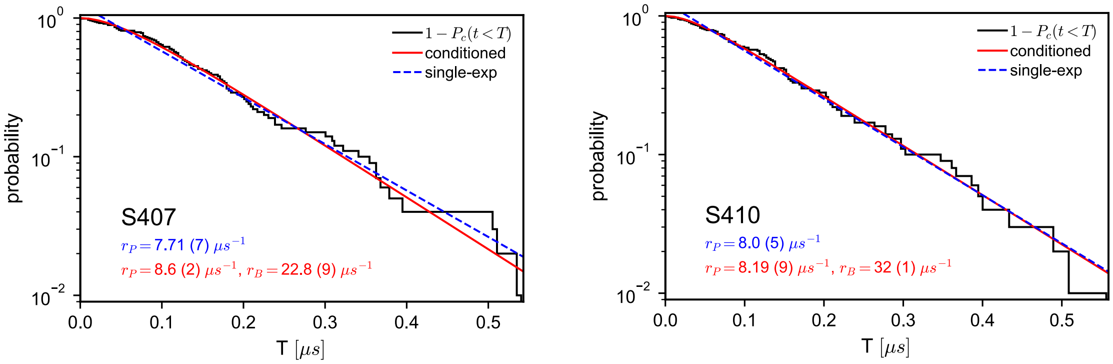

Supplementary Figure 10: Normalized inverse cumulative histograms of phosphorylation times (black solid lines) from 100 simulations with TDP-43 LCD and CK1δ folded domain and fit with simple single-exponential process (blue dotted lines, rate estimates in blue) and conditioned single-exponential process (red solid lines, rates estimates in red) for 8 different Ser residues.

Histograms of phosphorylation order

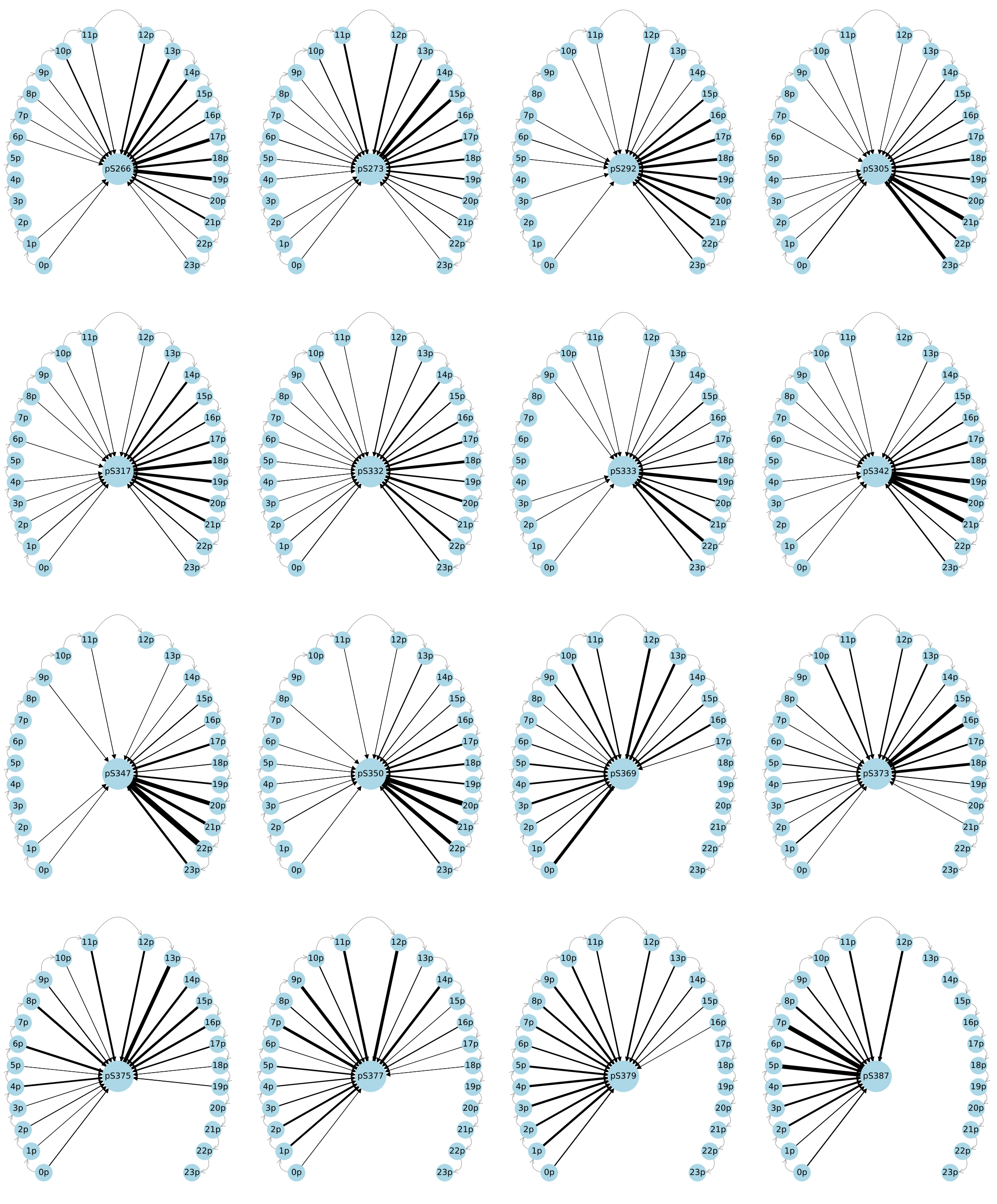

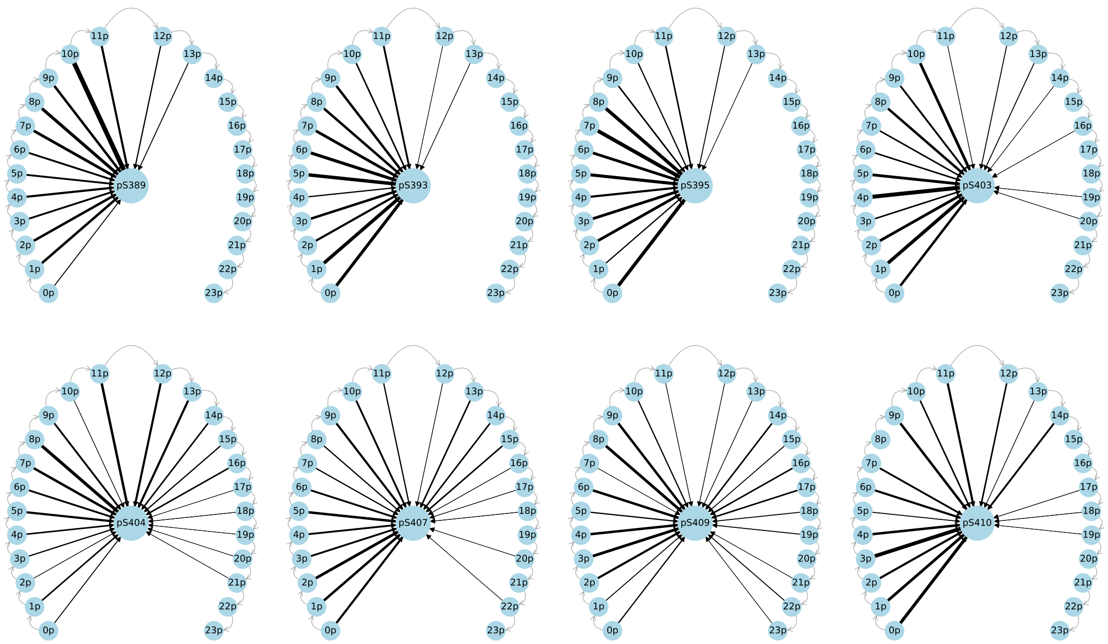

Supplementary Figure 11: Phosphorylation pattern representation for every Ser of TDP-43 LCD. The thickness of the arrows represent the percentage of simulations in which the Ser in the center of the graph was phosphorylated after n other Ser residues.

Phosphorylated TDP-43 LCD stays attached to CK1δ

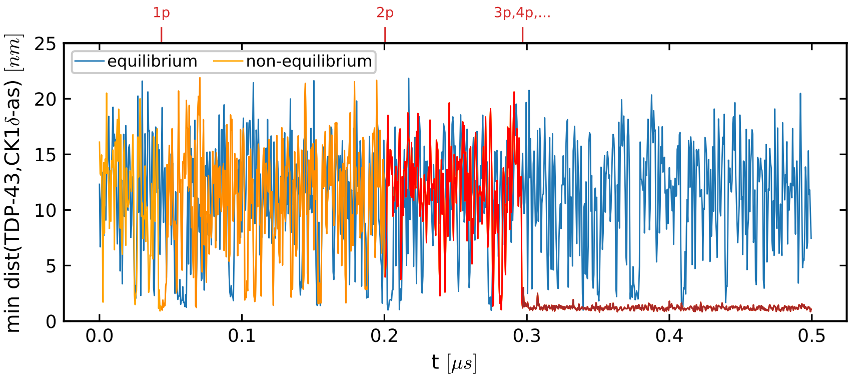

Supplementary Figure 12: Example trajectory of minimum distance between residues of TDP-43 LCD and the active site of CK1δ folded-domain in equilibrium simulation without phosphorylation (blue) and in non-equilibrium simulation (orange) in dilute concentration. The color of the non-equilibrium trajectory becomes darker after every phosphorylation event.

Determinants of the pattern of TDP-43 phosphorylation by CK1δ

Phosphorylation pattern for averaged-interaction chain

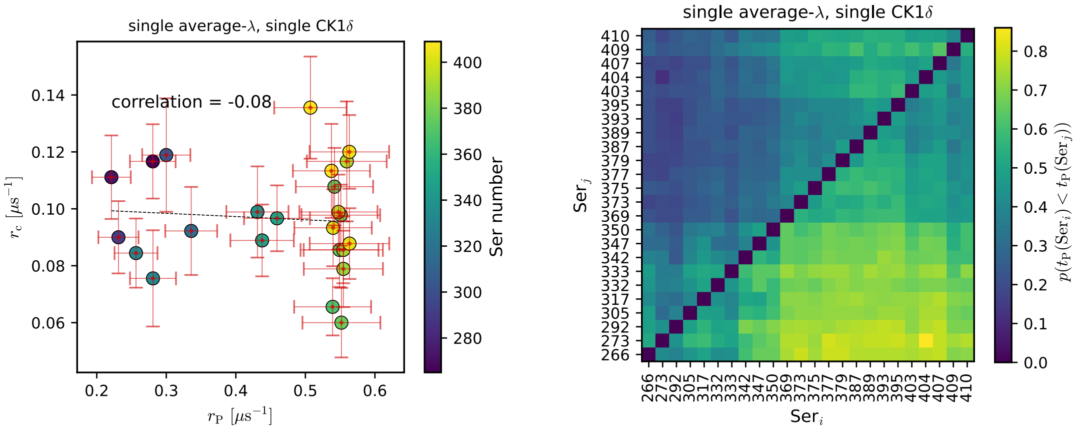

Supplementary Figure 13: Correlation plots of contact frequency in equilibrium r_c (mean +/- SEM from 30 replicas, Fig. 3i) and phosphorylation rates r_P (mean +/- SEM from 100 replicas, Fig. 3h) for simulations with averaged-interaction chain (average-λ sequence in Fig. 3g) and CK1δ folded-domain (left). Probability $p(t(\mathrm{Ser}_i) < t(\mathrm{Ser}_j))$ of Ser_i being phosphorylated ahead of Ser_j for system with averaged-interaction chain and CK1δ folded-domain from 100 trajectories (right).

Phosphorylation pattern for TDP-43 without charges

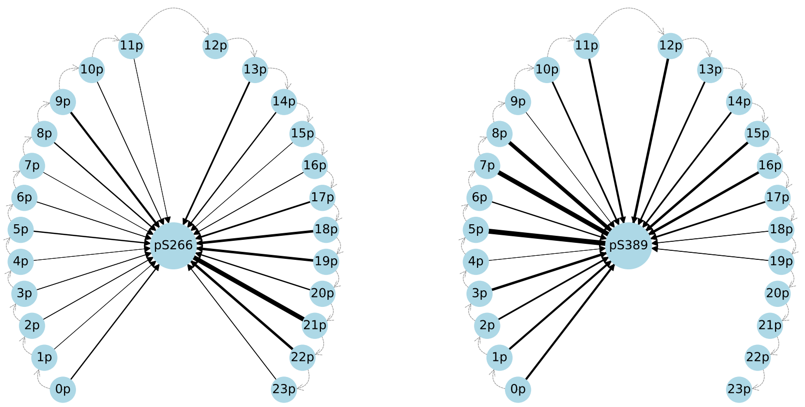

Supplementary Figure 14: Phosphorylation pattern representation for Ser266 and Ser389 of TDP-43 LCD without charges. The thickness of the arrows represent the percentage of simulations in which the Ser in the center of the graph was phosphorylated after n other Ser residues.

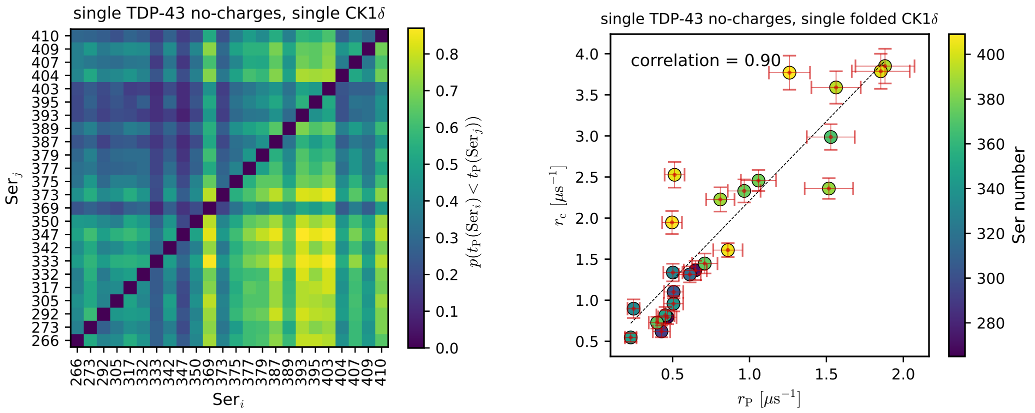

Supplementary Figure 15: Probability $p(t(\mathrm{Ser}_i) < t(\mathrm{Ser}_j))$ of Ser_i being phosphorylated ahead of Ser_j for system with TDP-43 without charges and CK1δ folded-domain from 100 trajectories (left). Correlation plots of contact frequency in equilibrium r_c (mean +/- SEM from 30 replicas, Fig. 3c) and phosphorylation rates r_P (mean +/- SEM from 100 replicas, Fig. 3b) for simulations with TDP-43 without charges and CK1δ folded-domain (right).

CK1δ phosphorylates TDP-43 in condensates

Phosphorylation rates at different times and pSer percentage

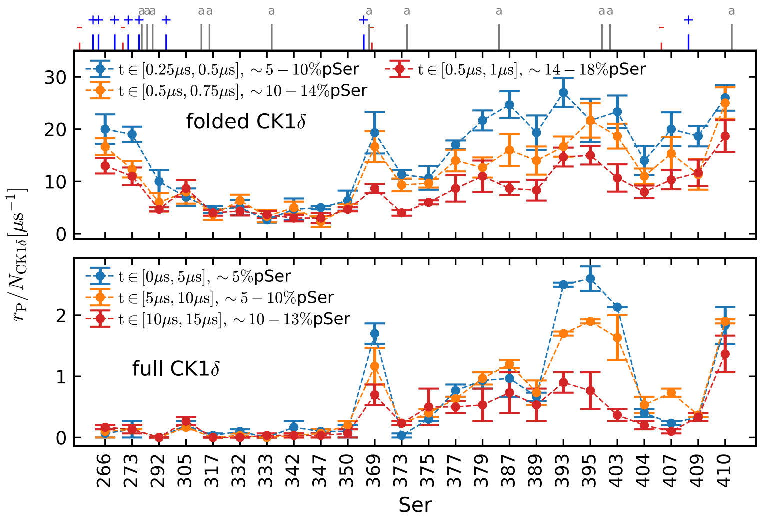

Supplementary Figure 16: Mean values +/- SEM of r_P for every Ser of TDP-43 LCD in condensate from 4 replicas with 3 CK1δ folded-domain (top panel) and 4 replicas with 3 full-length closed CK1δ (lower panel) for different parts of the trajectories. The ticks on top show the position of the charged (blue '+' positive, red '-' negative) and aromatic (grey 'a') residues.

Histogram of pSer per chain in dense phase

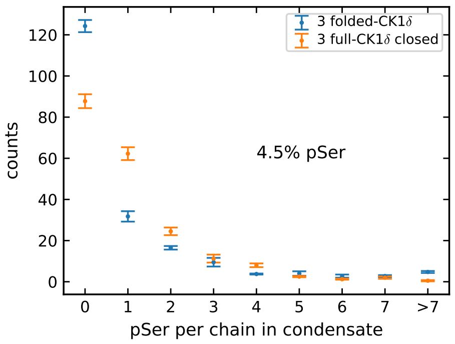

Supplementary Figure 17: Mean values +/- SEM of the histogram of the presence of phosphate per chain after 4.5% of Ser residues are phosphorylated, from 4 replicas of TDP-43 condensate simulations with 3 CK1δ folded-domain (blue) and with 3 full-length closed CK1δ (orange).

Density profile of Ser, pSer and CK1δ in dense phase

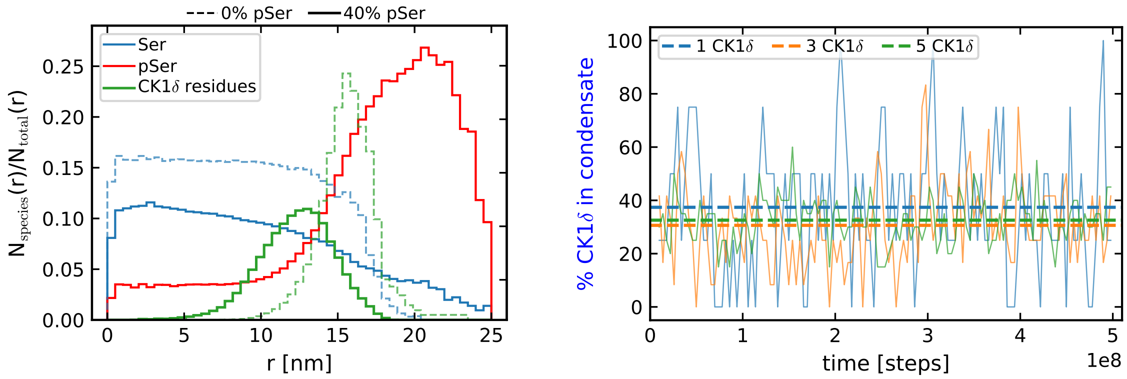

Supplementary Figure 18: Histogram of the number of Ser, pSer and CK1δ residues in condensate with bin counts normalized by the total number of beads in the bin (left). Only chains in the bigger cluster are taken into account for this plot. Percentage of CK1δ attached to the condensate in time for equilibrium simulations without phosphorylation with 1,3 and 5 CK1δ (right).

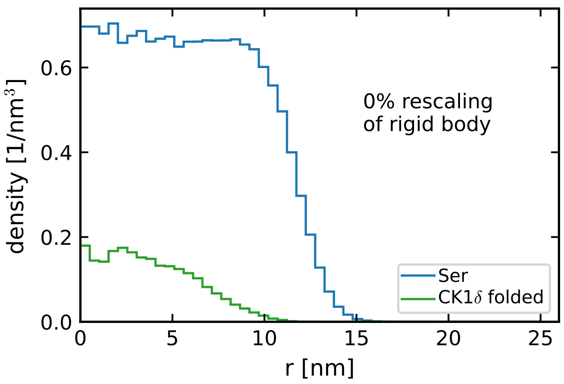

Supplementary Figure 19: Histogram of the number of Ser and CK1δ residues in condensate with bin counts normalized by the total number of beads in the bin for simulation without phosphorylations and without rescaling of CK1δ hydrophobic interactions.

CK1δ re-joins hyperphosphorylated condensate

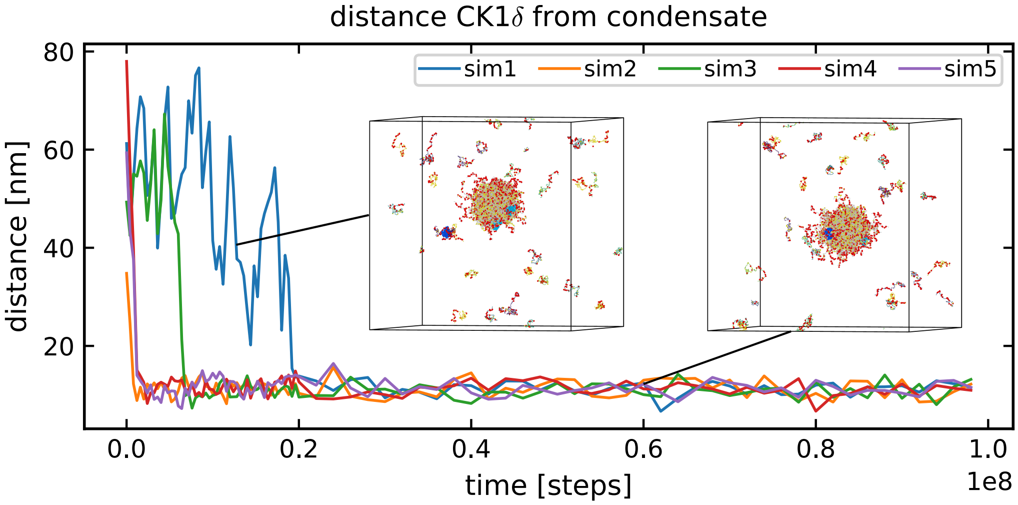

Supplementary Figure 20: Trajectory of distance between CK1δ and center of the TDP-43 LCD condensate from five equilibrium simulations at 40% pSer. CK1δ is placed outside of the condensate in the starting configuration. The insets show a snapshot with CK1δ (dark blue) still outside of the condensate and a snapshot with CK1δ in the condensate.

Distribution of pSer per chain in dilute phase

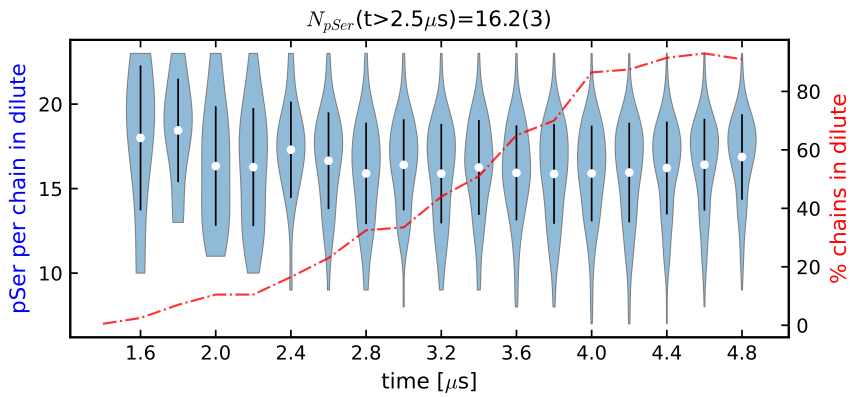

Supplementary Figure 21: Violin plot with distribution of the counts of pSer per TDP-43 chain in dilute phase at different times from the 200 TDP-43 chains in one single example trajectory. We show the mean of the distributions with white dots and the standard deviation with black errorbars. We compare them with the percentage of TDP-43 chains in dilute (red). By gathering the distributions for timesteps greater than 2.5×10^8 (when we have enough chains in dilute regime), we get that the mean number of pSer per chain in dilute is 16.2±0.3 .

Energy contributions from different pair potentials

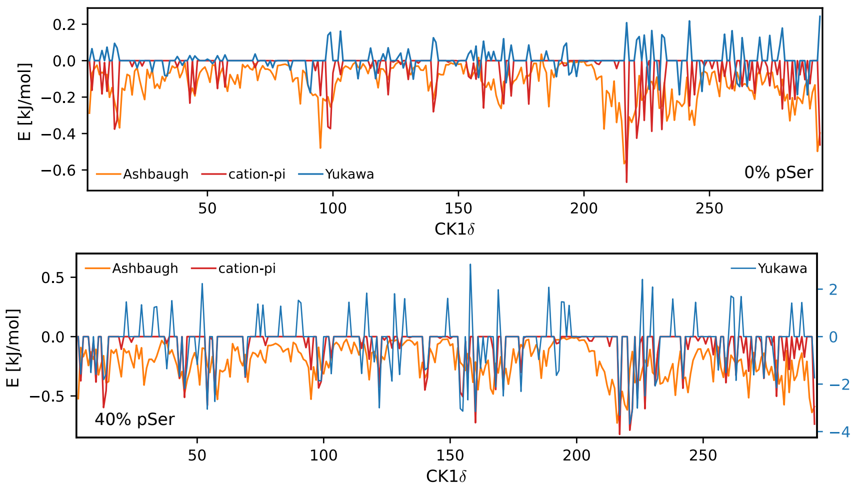

Supplementary Figure 22: Energy contribution per CK1δ residue for electrostatic interactions (Yukawa pair potential), hydrophobic interactions (Ashbaugh-Hatch pair potential) and cation-π interactions separately from equilibrium simulations both without pSer (up) and with 40% pSer (bottom) averaged from 30 uncorrelated frames.

Correlation plots in condensate

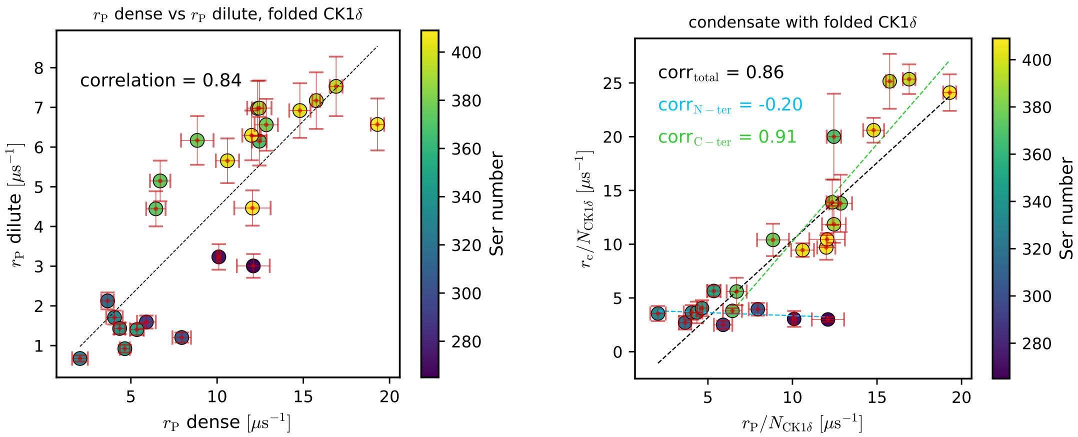

Supplementary Figure 23: Correlation plots for simulations with one CK1δ folded-domain of phosphorylation rates r_P in single-chain (y-axis, mean +/- SEM from 100 replicas, Fig. 3b) vs condensate simulations (x-axis, mean +/- SEM from 4 replicas, Fig. 4e) on the left and of contact frequency in equilibrium r_c (mean +/- SEM from 4 replicas, Fig. 4f) vs phosphorylation rates r_P (mean +/- SEM from 4 replicas, Fig. 4e) in condensate simulations on the right.

Effect of TDP-43 helical region

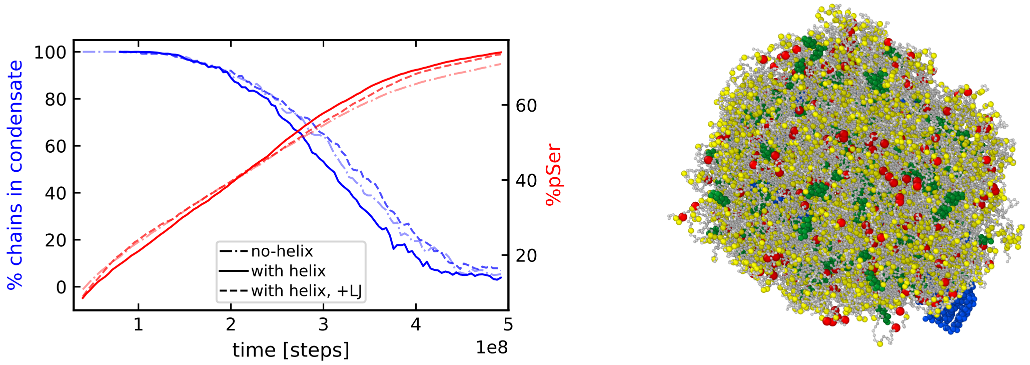

Supplementary Figure 24: On the left, percentage of TDP-43 chains in the condensate (blue, left y-axis) and percentage of phosphorylated Ser (red, right y-axis) in time for simulations with 5 CK1δ and with helical region of TDP-43 fixed as rigid body, both with (dashed lines) and without (continuous lines) additional helix-helix LJ potential. On the right, a snapshot a phosphorylated condensate (pSer in red, Ser in yellow) of 200 TDP-43 chains with preserved helical structure (green).

Role of CK1δ IDR in TDP-43 phosphorylation

Open vs closed configuration of CK1δ folded domain

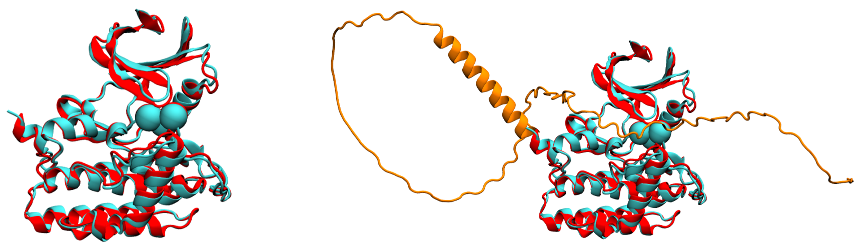

Supplementary Figure 25: Comparison of the folded domain structure for CK1δ from X-ray crystallography in blue (open configuration) and from AlphaFold2 prediction in red (closed configuartion). On the right we show the position of the disordered tail of CK1δ (orange) as predicted by AlphaFold2. The position of the active site Cα atoms is highlated with spherical beads.

Correlation plots for full-length CK1δ

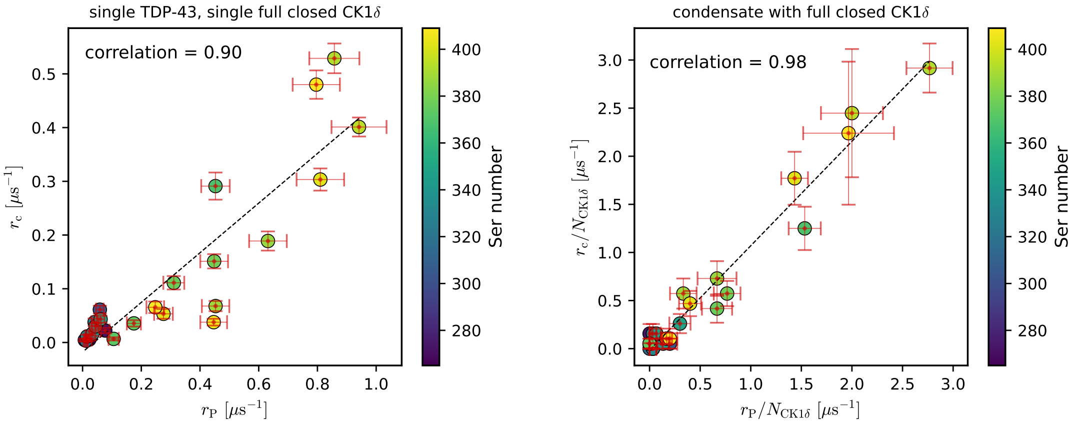

Supplementary Figure 26: Correlation plots for simulations with one full-length closed CK1δ of contact frequency in equilibrium vs phosphorylation rates in single-chain simulations (left, mean +/- SEM from 30 replicas for r_c, from 100 replicas for r_P, Fig. 5g,f) and condensate simulations (right, mean +/- SEM from 4 replicas for r_c and r_P, Fig. 5i,h).

Minimum distance of TDP-43 LCD from CK1δ active site

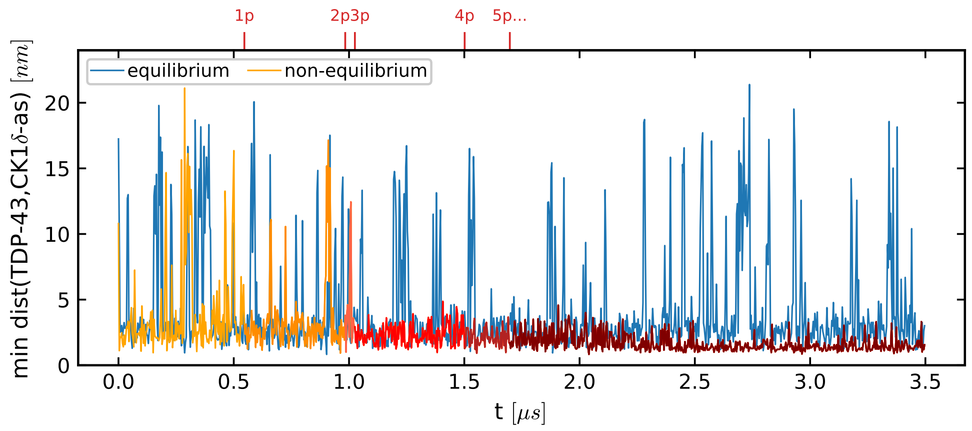

Supplementary Figure 27: Example trajectory of minimum distance between residues of TDP-43 LCD and the active site of full-length CK1δ in equilibrium simulation without phosphorylation (blue) and in non-equilibrium simulation (orange) in dilute concentration. The color of the non-equilibrium trajectory becomes darker after every phosphorylation event.

Minimum distance of CK1δ IDR from active site

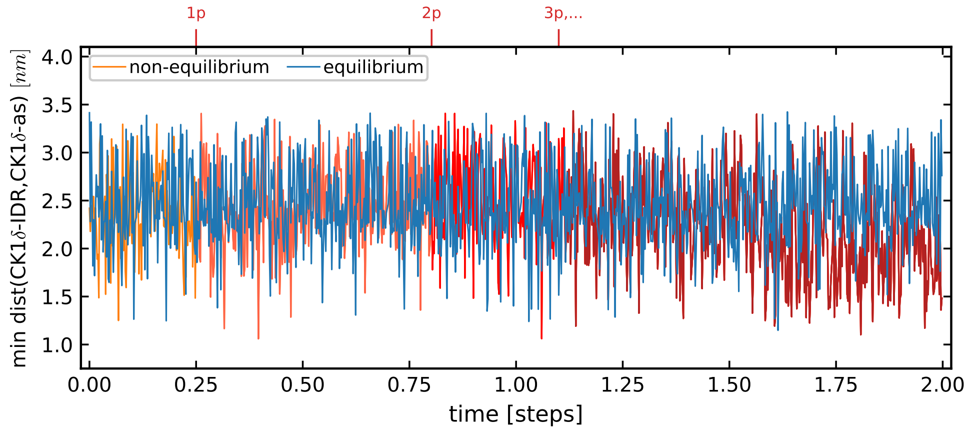

Supplementary Figure 28: Example trajectory of minimum distance between residues of full-length CK1δ IDR and its active site in equilibrium simulation without phosphorylation (blue) and in non-equilibrium simulation (orange) in dilute concentration. The color of the non-equilibrium trajectory becomes darker after every phosphorylation event.

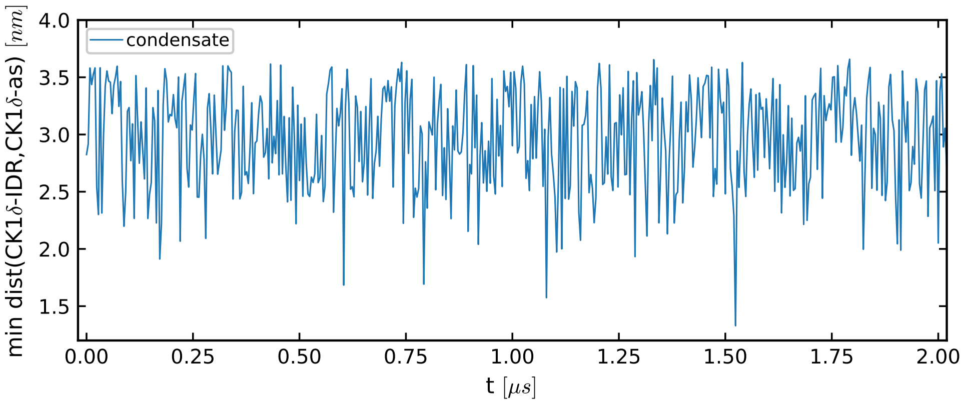

Supplementary Figure 29: Example trajectory of minimum distance between residues of full-length CK1δ IDR and its active site in condensate simulations.

Supplementary Tables

Thermodynamic consistency results

Ser	$-\Delta\mu_{\mathrm{P}}$ [kJ mol^{-1}]	$-\Delta\mu_{\mathrm{cycle}}$ [kJ mol^{-1}]	$-\Delta\mu_{cycle,3s}$ [kJ mol^{-1}]
Ser292	0	0.1 ± 0.6	0.1 ±0.6
	5	4.6 ± 0.9	4.6 ±0.9
	10	10.3 ± 0.8	10.3 ± 0.9
Ser317	0	-0.4 ± 0.5	-0.5 ± 0.5
	5	5.0 ± 0.7	4.9 ± 0.7
	10	10.3 ± 0.6	10.1 ± 0.8
Ser369	0	0.5 ± 0.7	0.5 ± 0.6
	5	5.0 ± 0.6	5.1 ± 0.5
	10	10.3 ± 0.7	10.2 ± 0.5
Ser387	0	0.0 ± 0.6	0.1 ± 0.4
	5	5.3 ± 0.6	5.2 ± 0.5
	10	9.3 ± 0.7	9.1 ± 0.7
Ser403	0	0.51 ± 0.26	0.52 ± 0.27
	5	4.87 ± 0.27	4.9 ± 0.3
	10	9.7 ± 1.0	9.5 ± 0.7
Ser409	0	0.0 ± 0.8	0.1 ± 0.7
	5	4.1 ± 0.7	4.1 ± 0.5
	10	10.3 ± 0.8	10.2 ± 0.8

Supplementary Table 1: Estimated $\Delta\mu_{\mathrm{cycle}}$ reported in Fig. 2E of the main text.

Ser	τ [steps]	$-\Delta\mu_P$ [kJ mol^{-1}]	$-\Delta\mu_{\mathrm{cycle}}$ [kJ mol^{-1}]
Ser403	1	0	0.45 ± 0.23
	10	0	0.51 ± 0.26
	20	0	0.57 ± 0.27
Ser403	1	-5	4.29 ± 0.21
	10	-5	4.86 ± 0.27
	20	-5	4.99 ± 0.34

Supplementary Table 2: Estimated $\Delta\mu_{\mathrm{cycle}}$ for Ser 403 and $\Delta\mu_{\mathrm{P}} = 0, -5$ kJ mol^{-1} for different lag times.

Simulation setup

Model	Sim. Type	$\Delta\mu_P$[kJ/mol]	TDP-43	CK1δ	Box Size	Sim. Time	Replicas
HPS (T=300K)	Thermo check (+Phos.) (+Reservoir)	0,-5,-10	WT (Ser292)	1 folded	50 nm	20 μs	1
		0,-5,-10	WT (Ser317)	1 folded	50 nm	20 μs	1
		0,-5,-10	WT (Ser369)	1 folded	50 nm	20 μs	1
		0,-5,-10	WT (Ser387)	1 folded	50 nm	20 μs	1
		0,-5	WT (Ser403)	1 folded	50 nm	25 μs	4
		-10	WT (Ser403)	1 folded	50 nm	20 μs	1
		0,-5,-10	WT (Ser409)	1 folded	50 nm	20 μs	1
	Δt_{MC}=100step	-5	WT (Ser403)	1 folded	50 nm	15 μs	1
	Δt_{MC}=400step	-5	WT (Ser403)	1 folded	50 nm	15 μs	1
	Δt_{MC}=1000step	-5	WT (Ser403)	1 folded	50 nm	15 μs	1
	Δt_{MC}=2000step	-5	WT (Ser403)	1 folded	50 nm	15 μs	1
mod. HPS (T=300K)	Single-chain (+Phos.)	-48	WT	1 folded	30 nm	2 μs	100
		-48	No-charge	1 folded	30 nm	2 μs	100
		-48	average-λ	1 folded	30 nm	4 μs	100
		-48	av-λ equi-Ser	1 folded	30 nm	4 μs	100
		-48	WT	1 full open	30 nm	2 μs	100
		-48	WT	1 full closed	30 nm	4 μs	100
	Single-chain (Equilibrium)	-	WT	1 folded	30 nm	15 μs	30
		-	No-charge	1 folded	30 nm	10 μs	30
		-	average-λ	1 folded	30 nm	30 μs	30
		-	av-λ equi-Ser	1 folded	30 nm	30 μs	30
		-	WT	1 full open	30 nm	10 μs	30
		-	WT	1 full closed	30 nm	30 μs	30
		-	WT helix	1 folded	30 nm	5 μs	10
	Condensate (+Phos.)	-48	200 WT	1 folded	100 nm	5 μs	4
		-48	200 WT	3 folded	100 nm	5 μs	4
		-48	200 WT	5 folded	100 nm	5 μs	4
		-48	200 WT	1 full closed	100 nm	3 μs	10
		-48	200 WT	2 full closed	100 nm	5 μs	4
		-48	200 WT	3 full closed	100 nm	5 μs	4
		-48	200 WT	1 full open	100 nm	5 μs	5
		-48	200 WT	3 full open	100 nm	5 μs	5
		-48	200 WT	5 full open	100 nm	5 μs	5
		-48	200 helix	5 folded	100 nm	5 μs	5
	Condensate (Equilibrium)	-	200 WT	1 folded	100	5 μs	4
		-	200 WT	3 folded	100	5 μs	4
		-	200 WT	5 folded	100	5 μs	4
		-	200 WT	1 full closed	100	5 μs	4
		-	200 WT	2 full closed	100	5 μs	4
		-	200 WT	3 full closed	100	5 μs	4
		-	200 WT	1 full open	100	1 μs	5
		-	200 WT	3 full open	100	1 μs	5
		-	200 WT	5 full open	100	1 μs	5
	Condensate (Eq. 40% pSer)	-	200 WT	5 folded	100 nm	1 μs	4
		-	200 WT	5 full open	100 nm	1 μs	4
		-	200 WT	5 folded (1-out)	100 nm	1 μs	5
	no-rescaling	-	200 WT	1 folded	100 nm	1 μs	1
CALVADOS3 (T=250K)	Single-chain (+Phos.)	-48	WT	1 folded	30 nm	2 μs	50
		-48	WT	1 full open	30 nm	2 μs	50

Supplementary Table 3: Summary of simulation setups for TDP-43 and CK1δ systems

Supplementary Notes

1. Transition probabilities and transition rates

We estimated the time-independent transition probability $T_{ij}(\tau)$, namely the probability of having the system in state j at time $t+\tau$ given that it was in state i at time t (for every t), using the non-reversible Maximum Likelihood Estimator[1,2]:

$$T_{ij}(\tau) = \frac{c_{ij}(\tau)}{\sum_k c_{ik}(\tau)} \tag{1}$$

where $c_{ij}(\tau)$ is the count of transitions from i to j after a lag time τ. For a Markov process, the transition probability matrix $\mathbf{T}(\tau)$ can be expressed in terms of transition rates matrix $\mathbf{k}$ with continuous time as:

$$\mathbf{T} = \exp(\mathbf{k}\tau). \tag{2}$$

For small τ compared to the system timescales, Equation 2 can be approximated as

$$\mathbf{T} \simeq \mathbb{1} + \tau\mathbf{k} \quad \Rightarrow \quad k_{ij} \simeq \frac{T_{ij}}{\tau}, \quad \forall i, j \text{ such that } i \neq j. \tag{3}$$

In such case, using T_{ij} or k_{ij} to compute $\Delta\mu_{\text{cycle}}$ does not change the result, since the factor $1/\tau$ would be canceled out in the ratio in Equation 3 of the main text.

As an example, for the simulations with reactive Ser 403 and $\Delta\mu_{\text{P}} = -5$ kJ mol^{-1}, we computed T_{ij} with lag time $\tau = 10$ Markov chain steps (or 1 ns in simulation time). Discretizing the trajectory into 3 Markov states (state 1 with unbound CK1δ and TDP-43, state 2 with bound configuration and unphosphorylated Ser 403 and state 3 with bound configuration and phosphorylated Ser 403) leads to 2 implied timescales that are much larger than the the lag time τ (Fig. 2, Supplementary Fig. 3). The estimated transition probability matrix is

$$\mathbf{T} = \begin{bmatrix} 0.97706502 & 0.01522616 & 0.00770882 \\ 0.03748648 & 0.92563442 & 0.0368791 \\ 0.02103749 & 0.00579359 & 0.97316892 \end{bmatrix} \tag{4}$$

while the transition rates matrix is

$$\mathbf{k} = \begin{bmatrix} -0.02293498 \text{ ns}^{-1} & 0.01522616 \text{ ns}^{-1} & 0.00770882 \text{ ns}^{-1} \\ 0.03748648 \text{ ns}^{-1} & -0.07436558 \text{ ns}^{-1} & 0.0368791 \text{ ns}^{-1} \\ 0.02103749 \text{ ns}^{-1} & 0.00579359 \text{ ns}^{-1} & -0.02683108 \text{ ns}^{-1} \end{bmatrix} \tag{5}$$

where for the diagonal elements we used the property of the rate matrices $k_{ii} = -\sum_{j\neq i} k_{ij}$.

2. Detailed balance breaking

The transitions $1 \rightleftharpoons 2$, $3 \rightleftharpoons 4$ (the binding/unbinding of the enzyme with TDP-43 or phosphorylated TDP-43) are determined through the MD simulation, which samples from the standard Boltzmann distribution $p_i = \exp(-\beta\mathcal{H}_i)$, with $\mathcal{H}_i$ the total energy of the configuration i. In a similar way, $4 \rightleftharpoons 1$, i.e. the reservoir exchange step, is determined by the acceptance probability of the Metropolis step without chemical fuel and the probability of attempting the MC move. The probability of attempting the MC move, in this case, depends again on the probability of having enzyme and

substrate far enough (determined through MD simulation) and the fixed rate at which we check if this condition is fulfilled in our simulation (main text, Methods). Thus, we expect for those 3 transitions to not be driven by any external energy source and to have a rate ratio k_{ij}/k_{ji} fixed by the free energies of the states through

$$\frac{k_{ij}}{k_{ji}} = \exp\left(-\beta \Delta F_{ij}\right) \tag{6}$$

where ΔF_{ij} is the free energy difference between state j and i.

Instead, the acceptance probability of the MC phosphorylation step in Equation 2 of the main text leads to a ratio of reaction rates $k_{AB}/k_{BA} = \exp(-\beta\Delta U_P - \beta\Delta\mu_{\mathrm{P}})$ $2 \rightleftharpoons 3$, where A and B are respectively the configuration with contact between Ser and active site and the one with pSer in the place of Ser. Given that the probability of having a contact between Ser (or pSer) and active site is determined by the MD simulation and that the attempt rate of the MC move is again fixed in the simulation, we expect a rate ratio

$$\frac{k_{23}}{k_{32}} = \exp\left(-\beta \Delta F_{23} - \beta\Delta\mu_{\mathrm{P}}\right) \tag{7}$$

where the term with $\Delta\mu_{\mathrm{P}}$ comes from the ratio of the reaction rates. In this sense, despite the Metropolis step is built in such a way to satisfy the detailed balance condition, the $\Delta\mu_{\mathrm{P}}$ added in the acceptance ratio breaks detailed balance once the algorithm is coupled to equilibrium MD simulations.

By using Supplementary Equation 6 and 7, we can simplify Equation 3 in the main text and show that $\Delta\mu_{\mathrm{cycle}} = \Delta\mu_{\mathrm{P}}$:

$$\begin{aligned}\Delta\mu_{\mathrm{cycle}} &= -\frac{1}{\beta}\ln\left(\frac{k_{12}}{k_{21}}\right) - \frac{1}{\beta}\ln\left(\frac{k_{23}}{k_{32}}\right) - \frac{1}{\beta}\ln\left(\frac{k_{34}}{k_{43}}\right) - \frac{1}{\beta}\ln\left(\frac{k_{41}}{k_{14}}\right) = \\ &= \Delta F_{12} + \Delta F_{23} + \Delta\mu_{\mathrm{P}} + \Delta F_{34} + \Delta F_{41} = \Delta\mu_{\mathrm{P}}.\end{aligned} \tag{8}$$

3. Thermodynamic consistency results and additional robustness checks

We repeated the estimate of $\Delta\mu_{\mathrm{cycle}}$ using a 3-states MSM, in which the unbound states 1 and 4 are merged into the new state 1. The results are in agreement with the 4-states MSM (Supplementary Table 1, Supplementary Fig. 3). Indeed, the transition between state 1 and 4 has a very high rate and can be associated with the smallest implied timescale, that is lower than the lag time for $\tau = 10$ Markov chain steps or larger.

We also checked the reliability of VAMPnet by using considerably more input distances (4620 distances) and an additional layer, again for the case of reactive Ser 403 and $\Delta\mu_{\mathrm{P}} = -5$ kJ mol^{-1} (Methods in main text). The estimated $\Delta\mu_{\mathrm{cycle}}$ with the new version of VAMPnet is $\Delta\mu_{\mathrm{cycle}} = 4.7 \pm 0.6$ kJ mol^{-1} (implied timescales and CK test in Supplementary Fig. 4).

4. Chemical potential difference in a phosphorylation cycle

The chemical reaction difference in a reaction in units of RT is given by the logarithm of the product to substrate concentration ratio: In the phosphorylation-dephosphorylation cycle, the chemical reactions involved are the two following ones:

$$\mathrm{Ser} + \mathrm{ATP} \rightleftharpoons \mathrm{pSer} + \mathrm{ADP}\ ; \qquad \mathrm{pSer} \rightleftharpoons \mathrm{Ser} + \mathrm{P}_i$$

The chemical potential differences for the two reactions are

$$\Delta\mu_P = \Delta\mu_P^0 + RT\log\left(\frac{[\text{TDP-43*}][\text{ADP}]}{[\text{TDP-43}][\text{ATP}]}\right) = RT\log\left(\frac{[\text{TDP-43*}][\text{ADP}]}{[\text{TDP-43}][\text{ATP}]}\frac{[\text{TDP-43}]_{eq}[\text{ATP}]_{eq}}{[\text{TDP-43*}]_{eq}[\text{ADP}]_{eq}}\right) \tag{9}$$

$$\Delta\mu_D = \Delta\mu_D^0 + RT\log\left(\frac{[\text{TDP-43}][\text{P}_i]}{[\text{TDP-43*}]}\right) = RT\log\left(\frac{[\text{TDP-43}][\text{P}_i]}{[\text{TDP-43*}]}\frac{[\text{TDP-43*}]_{eq}}{[\text{TDP-43}]_{eq}[\text{P}_i]_{eq}}\right) \tag{10}$$

The total amount of chemical driving will be:

$$\begin{aligned}\Delta\mu_{\circlearrowleft} &= \Delta\mu_P + \Delta\mu_D = \\ &= RT\log\left(\frac{[\text{TDP-43*}][\text{ADP}]}{[\text{TDP-43}][\text{ATP}]}\frac{[\text{TDP-43}]_{eq}[\text{ATP}]_{eq}}{[\text{TDP-43*}]_{eq}[\text{ADP}]_{eq}}\frac{[\text{TDP-43}][\text{P}_i]}{[\text{TDP-43*}]}\frac{[\text{TDP-43*}]_{eq}}{[\text{TDP-43}]_{eq}[\text{P}_i]_{eq}}\right) = \\ &= RT\log\left(\frac{[\text{ADP}][\text{P}_i]}{[\text{ATP}]}\frac{[\text{ATP}]_{eq}}{[\text{ADP}]_{eq}[\text{P}_i]_{eq}}\right) = \Delta\mu_{ATP}^0 + RT\log\left(\frac{[\text{ADP}][\text{P}_i]}{[\text{ATP}]}\right)\end{aligned} \tag{11}$$

Note that $\Delta\mu_{\circlearrowleft}$ is equal to the chemical potential difference for the ATP hydrolysis reaction ATP $\rightleftharpoons$ ADP + P_i, for which the equilibrium value in standard conditions is $\Delta\mu_{ATP}^0 = -7.3$ kcal mol^{-1}.

5. Binding free energy

We computed the binding free energy ΔG_{bind} between 1) CK1δ folded-domain and wild type TDP-43 LCD, 2) full-length CK1δ and wild type TDP-43 LCD, 3) CK1δ folded-domain and triple phosphorylated TDP-43 LCD (pSer 395, pSer 403, pSer 410). For the first 2 cases, we used the data from the equilibrium simulations without phosphorylation step (450 μs for case 1) and 900 μs for case 2)), while for case 3) we collected 20 μs of simulation time.

The binding free energy is estimated as

$$\Delta G_{bind} = -RT\ln\left(\frac{T_{u,b}(\tau)}{T_{b,u}(\tau)}\right) \tag{12}$$

where $T_{u,b}(\tau)$ is the probability to have a bound state at time $t+\tau$, given an unbound state at time t, and $T_{b,u}(\tau)$ is the probability to have an unbound state at time $t+\tau$, given a bound state at time t. The lag time considered here is the time difference between 2 consecutive snapshots, i.e. $\tau = 5\times10^4$ MD steps. In order to get the transition probabilities, we discretize the simulation trajectories in bound and unbound state using VAMPnet (in the same way as in Fig. 2, as explained in Methods) and estimate $T_{u,b}$ and $T_{b,u}$ using a Maximum Likelihood estimator for MSM[1].

Supplementary Methods

Residue-level coarse-grained models with implicit solvent

For our coarse-grained simulations we employed the hydrophobicity scale (HPS) model[3], a modification of it[4] and CALVADOS3 to check the reliability of our results[5]. The original HPS model was fitted with IDPs data and considers proteins as fully flexible chains. In order to have a more realistic representation of the enzyme CK1δ, we decided to employ also a modified version of it that takes into account the presence of folded domains. In both models every residue type is represented with a particle of Lennard-Jones (LJ) size σ, charge q, mass m and hydropathy scale parameter λ. For the HPS model, the pair potential has 3 contributions

$$U^{ij}_{\text{HPS}} = U^{ij}_{\text{bond}} + U^{ij}_{\text{electrostatic}} + U^{ij}_{\text{hydrophobicity}}, \tag{13}$$

while the modified HPS model has one more contribution to enhance cation-π interactions:

$$U^{ij}_{\text{mHPS}} = U^{ij}_{\text{bond}} + U^{ij}_{\text{electrostatic}} + U^{ij}_{\text{hydrophobicity}} + U^{ij}_{\text{cation}-\pi}. \tag{14}$$

The bonded interactions are described by an harmonic potential

$$U^{ij}_{\text{bond}} = \frac{1}{2}k(r_{ij} - r_0)^2 \tag{15}$$

with r_{ij} the distance between the neighboring residues i and j, spring constant $k = 8360$ kJ mol^{-1} nm^{-2} and equilibrium bond length $r_0 = 0.381$ nm.

The interactions between non-bonded residues are modeled through the Ashbaugh-Hatch potential

$$U^{ij}_{\text{hydrophobicity}} = \begin{cases} U^{ij}_{\text{LJ}} + \epsilon(1-\lambda_{ij}), & \text{if } r_{ij} \le 2^{1/6}\sigma_{ij} \\ \lambda_{ij} U^{ij}_{\text{LJ}}, & \text{if } r_{ij} > 2^{1/6}\sigma_{ij} \end{cases} \tag{16}$$

where $\sigma_{ij} = (\sigma_i + \sigma_j)/2$, $\lambda_{ij} = (\lambda_i + \lambda_j)/2$ and U^{ij}_{LJ} is the standard Lennard-Jones potential

$$U^{ij}_{\text{LJ}} = 4\epsilon\left[\left(\frac{\sigma_{ij}}{r_{ij}}\right)^{12} - \left(\frac{\sigma_{ij}}{r_{ij}}\right)^{6}\right]. \tag{17}$$

The interaction is truncated at a cutoff distance of 2 nm. The parameter ϵ expresses the strength of the Lennard-Jones interaction and it is fixed to $\epsilon = 0.8368$ kJ mol^{-1}, value fitted with experimental R_g from single IDP chains[3], while the hydrophaty scale parameter λ_{ij} scales down the interaction for distances larger than the minimum of U^{ij}_{LJ} and goes from 0 (fully hydrophilic case, no attraction between residues) to 1 (fully hydrophobic case, the interaction becomes a standard LJ). Phosphorylated Ser residues are modelled as described by Perdikari et al[6].

Charged residues experience also salt-screened electrostatic interactions, which are modeled using a Yukawa/Debye-Hückel potential

$$U_{\text{electrostatic}} = \frac{q_i q_j e^2}{4\pi\epsilon_0\epsilon_r r}\exp(-r/r_D) \tag{18}$$

where we used a Debye screening length $r_D = 1.0nm$ for an ionic strength of approximately 100 mM and a relative dielectric constant of the water solvent $\epsilon_r = 80$, following the ones of the original HPS model[3]. In this case the cutoff distance is 3.5 nm.

For the modified HPS model, we added another LJ potential only between cation-π pairs (Arg/Lys with Phe/Trp/Tyr), as proposed by Das et al.[7]:

$$U^{ij}_{\text{cation}-\pi} = U^{ij}_{\text{LJ}} \text{ , with } \epsilon_{\text{cation}-\pi} = 3.138 \frac{kJ}{mol}. \tag{19}$$

Also in this case the cutoff is 2 nm.

In the modified HPS model, the dynamics of folded domains follows the one of a rigid body. Moreover, the parameter λ in $U^{ij}_{\text{hydrophobicity}}$ and $\epsilon_{\text{cation}-\pi}$ in $U^{ij}_{\text{cation}-\pi}$ are scaled down by 30% for pair interactions involving residues of the folded domains, as suggested by Krainer et al.[8].

The CALVADOS3 model uses the same pair potentials of the standard HPS model, but with different λ parameters[5]. For the phosphorylated Ser residues in CALVADOS3 simulations, we used the λ parameter of the aspartic acid (close to 0). In the bonded interactions, the spring constant is fixed to $k = 8033$ kJ mol^{-1} nm^{-2}. The electrostatic interaction cutoff is increased to 4 nm. Folded domains are modeled using an elastic network. Beads with pair distance smaller than 0.9 nm are connected though harmonic potentials with spring constant 700 kJ mol^{-1} nm^{-2}. The other setup parameters are consistent with the ones from the standard HPS model.

The Ashbaugh-Hatch pair potential for the non-bonded interactions is available at `https://github.com/ezippo/ashbaugh_plugin` as a HOOMD-blue plugin. The code used for the simulations is available at `https://github.com/ezippo/hoomd3_phosphorylation`. The code used for this manuscript can be also found in the Zenodo repositiories[9–11].

Additional thermodynamic validation simulations

We show in Supplementary Fig. 1 the dependence of transition rates of 4-state MSM (sketch in Fig. 1 in the main text) and their ratios from the choice of the MC move attempt rate. We ran simulations with reactive Ser 403 and $\Delta\mu_{\text{P}} = -5$ kJ mol^{-1} using MC attempt rates 100, 200 (from thermodynamic validation simulations), 400, 1000 and 2000. For each attempt rate, we collected 15 μs of simulation time for each MC move attempt rate.

Additional CALVADOS3 simulations

To ensure that our results are not overly dependent on the choice of the simulation model, we repeated them with CALVADSOS3 force field[5,12]. We computed the phosphorylation rates r_{P} (Supplementary Fig. 8) from 50 replicas of 2 μs each, both for the case of single TDP-43 LCD single CK1δ folded domain and of single TDP-43 LCD single full-length open CK1δ. The temperature in CALVADOS3 simulation is lowered to 250 K in order to keep the ratio with the critical temperature roughly constant:

$$\frac{T(\text{CALVADOS3})}{T_c(\text{CALVADOS3})} \simeq \frac{T(\text{mod.HPS})}{T_c(\text{mod.HPS})}.$$

Simulations with preserved helix

To ensure that our results are not overly dependent on the choice of the simulation model, we also investigated the effect of explicitly modeling the C-terminal helix (residues 319-341) of TDP-43 LCD. In order to model the C-terminal helix, we fixed the relative positions of residues 320-332 as a rigid body. We computed the rate of contact formation between Ser residues of TDP-43

LCD and CK1δ active site (Supplementary Fig. 9) from a total of 100 μs of equilibrium simulation without phosphorylations. We also repeated the condensate simulation with 200 TDP-43 LCD with fixed helix and 5 CK1δ folded domain. In this case, we increased the well depth of the helix-helix interactions by adding a LJ pair potential between residues belonging to the helices of different chains. The depth of the LJ potential (ϵ parameter) is fixed to 1 tenth of k_BT. We simulated 5 replicas of 5 μs each, both with and without the additional helix-helix LJ potential.

Supplementary References

1. Trendelkamp-Schroer, B., Wu, H., Paul, F. & Noé, F. Estimation and uncertainty of reversible Markov models. *The Journal of Chemical Physics* **143,** 174101 (2015).
2. Hoffmann, M. *et al.* Deeptime: a Python library for machine learning dynamical models from time series data. *Machine Learning: Science and Technology* (2021).
3. Dignon, G. L., Zheng, W., Kim, Y. C., Best, R. B. & Mittal, J. Sequence determinants of protein phase behavior from a coarse-grained model. *PLoS Comput. Biol.* **14,** e1005941 (2018).
4. Tejedor, A. R., Garaizar, A., Ramírez, J. & Espinosa, J. R. 'RNA modulation of transport properties and stability in phase-separated condensates. *Biophysical Journal* **120,** 5169–5186 (2021).
5. Cao, F., von Bülow, S., Tesei, G. & Lindorff-Larsen, K. A coarse-grained model for disordered and multi-domain proteins. *Protein Science* **33,** e5172 (2024).
6. Perdikari, T. M., Jovic, N., Dignon, G. L., Kim, Y. C., Fawzi, N. L. & Mittal, J. A predictive coarse-grained model for position-specific effects of post-translational modifications. *Biophys. J.* **120,** 1187–1197 (2021).
7. Das, S., Lin, Y.-H., Vernon, R. M., Forman-Kay, J. D. & Chan, H. S. Comparative roles of charge, π, and hydrophobic interactions in sequence-dependent phase separation of intrinsically disordered proteins. *Proceedings of the National Academy of Sciences* **117,** 28795–28805 (2020).
8. Krainer, G. *et al.* Reentrant liquid condensate phase of proteins is stabilized by hydrophobic and non-ionic interactions. *Biophysical Journal* **120,** 28a (2021).
9. Zippo, E. *Supporting Data: Molecular simulations of enzymatic phosphorylation of disordered proteins and their condensates* 2024.
10. Zippo, E. *ezippo/ashbaugh_plugin: Molecular simulations of enzymatic phosphorylation of disordered proteins and their condensates* version v1.0.0. 2025.
11. Zippo, E. *ezippo/hoomd3_phosphorylation: Molecular simulations of enzymatic phosphorylation of disordered proteins and their condensates* version v2.0.1. 2025.
12. Tesei, G. & Lindorff-Larsen, K. Improved predictions of phase behaviour of intrinsically disordered proteins by tuning the interaction range [version 2; peer review: 2 approved]. *Open Research Europe* **2** (2023).
