# Supplementary material for: Molecular simulations of enzymatic phosphorylation of disordered proteins and their condensates
Source: Nat Commun. 2025 May 19;16:4649. doi: 10.1038/s41467-025-59676-4 (PMC12089381; doi:10.1038/s41467-025-59676-4)
Supplement: Supplementary file 2 — Description of Additional Supplementary Files [file 41467_2025_59676_MOESM2_ESM.pdf]

Description of Additional Supplementary Files

Supplementary Movie 1:
Movie from simulation of single TDP-43 LCD chain and single CK1δ folded domain (blue) with phosphorylation step and reservoir exchange step in cubic box of 50nm side length using HPS model. In this simulation, the only phosphosite is Ser 403 (red when phosphorylated) and $\Delta\mu_P$=-5 kJ mol^{-1} (simulation used in Fig.2).

Supplementary Movie 2:
Movie from simulation of single TDP-43 LCD chain and single CK1δ folded domain (blue) with phosphorylation step in cubic box of 30nm side length using modified HPS model. Serines in yellow, phospho-serines in red. Ser 410 is highlighted with a larger bead (simulation used in Fig.3).

Supplementary Movie 3:
Movie of dissolution of TDP-43 condensate through enzymatic hyperphosphorylation from 5μs long simulation of 200 TDP-43 LCD chains and 5 CK1δ folded-domain (blue) with phosphorylation step in cubic box of 100nm side length using modified HPS model. Serines in yellow, phospho-serines in red (simulation used in Fig.4).

Supplementary Movie 4:
Movie from simulation of single TDP-43 LCD chain and single full-length closed CK1δ (blue, IDR in light blue) with phosphorylation step in cubic box of 30nm side length using modified HPS model. Serines in yellow, phospho-serines in red (simulation used in Fig.5).

Supplementary Movie 5:
Movie from simulation of 200 TDP-43 LCD chains and 3 full-length closed CK1δ (blue, IDR in light blue) with phosphorylation step in cubic box of 100nm side length using modified HPS model. Serines in yellow, phospho-serines in red (simulation used in Fig.5).

Supplementary Movie 6:
Movie from simulation of 200 TDP-43 LCD chains and 5 CK1δ folded domain in equilibrium without phosphorylation step in cubic box of 100nm side length using modified HPS model. The simulation starts from a frame with 40% of phosphorylated serines (red beads), one CK1δ chain in dilute phase (dark blue) and four CK1δ chains in condensate (light blue) (simulation used in Supplementary Fig.20).
